# Comparative Study of Two Different TiO_2_ Film Sensors on Response to H_2_ under UV Light and Room Temperature

**DOI:** 10.3390/s16081249

**Published:** 2016-08-08

**Authors:** Xiaoying Peng, Zhongming Wang, Pan Huang, Xun Chen, Xianzhi Fu, Wenxin Dai

**Affiliations:** 1 State Key Laboratory of Photocatalysis on Energy and Environment, Research Institute of Photocatalysis, Fuzhou University, Fuzhou 350002, China; n130420102@fzu.edu.cn (X.P.); n141320126@fzu.edu.cn (Z.W.); chenxun@fzu.edu.cn (X.C.); xzfu@fzu.edu.cn (X.F.); 2 College of Chemistry, Fuzhou University, Fuzhou 350108, China; n130420085@fzu.edu.cn

**Keywords:** semiconductor gas sensor, in-situ fabrication, photo-response, photo-electrochemical measurement, electron transfer

## Abstract

An anatase TiO_2_ film sensor was prepared by a facile in-situ method on the interdigitated Au electrode deposited on the alumina substrate. The structure, morphology and the optical properties of the in-situ TiO_2_ film sensor were characterized by X-ray diffraction, Scanning Electron Microscopy, and UV-vis diffuse reflectance spectra. The photo-assisted gas sensitivities of the prepared film towards H_2_ gas were evaluated at room temperature in N_2_ and synthetic air atmospheres. As compared to TiO_2_ film sensor prepared by drop-coating method, this in-situ TiO_2_ film sensor exhibited a more compact structure composed of uniform TiO_2_ microspheres as well as a better gas sensitivity towards H_2_ under UV irradiation, especially in synthetic air. The photo-electrochemical measurements suggest that these improvements may be associated with the efficient charge transfer in the TiO_2_ interface induced by the TiO_2_ microsphere structure. This study might offer a feasible approach to develop photo-assisted gas sensors at ambient temperature.

## 1. Introduction

In the recent years, gas sensors have been widely investigated due to the increasing demand for their practical applications notably in the detection of dangerous/poisonous gases, medical practice, security, industrial manufacture, and bioengineering [1,2,3,4,5,6]. Metal oxide gas sensors play important roles in the gas sensing research because of their prominent advantages of high sensitivity, good stability, and fast response. In particular, TiO_2_ metal oxides were found very promising in numerous application areas including photovoltaics, photocatalysis, catalysis, and sensors [7,8,9,10,11]. When TiO_2_ oxides are utilized as thermal sensors, they might be limited by the high operating temperatures, issues not well accepted from the perspectives of energy cost and the environmental safety. Furthermore, their repeatability and stability could decrease after exposure to reducing or oxidizing gases [12,13,14]. With this respect, a number of studies show that doping the metal as well as controlling its morphology could improve the sensitivities of the metal oxides gas sensors at low temperatures [15,16,17,18,19,20,21,22,23,24]. For instance, Pb-doped TiO_2_ nanofibers investigated by Moon et al. [15] yielded highly sensitive NO_2_ sensors with remarkably stable responses to NO_2_ gas towards concentrations reaching as low as 0.16 ppm at 180 °C. Some studies showed that doping TiO_2_ with metal ions such as Ta, La, Nb, and Cr is feasible and results in improved O_2_ sensitivities [16,17,18,19,20]. In addition, Noble metals like Pt, Pd, Au, and Ag have been utilized to reduce the operation temperatures through effective improvements of the interaction between TiO_2_ surface and the gas molecules [21,22,23,24]. Other studies addressed some limitations of the gas sensors by improving the structure and morphology of the gas sensor films. For example, a cauliflower-like structure of λ-Al_2_O_3_ films was achieved by chemical vapor deposition and showed an improved response to CO_2_ gas [25]. Sonker et al. [26] successfully prepared a nano-petal like NO_2_ sensor through a cheap and easy chemical route. Plecenik et al. [21] constructed a highly-sensitive room-temperature semiconductor gas sensor based on nanoscale Pt-TiO_2_-Pt sandwich.

On the other hand, considerable efforts have recently been devoted to enhancing the gas sensitivity of the semiconductor sensors and decrease their response temperatures by the use of light radiation [27,28], including metal oxides [29,30,31,32]. With the introduction of light (exciting the response of semiconductor), the gas sensing operating temperature could be reduced to a great extent, and was even applicable at room temperature. For example, we have also previously reported a ZnO gas sensor for acetone and ethylene under visible light irradiation at room temperature [31]. Moreover, the photo-assisted response properties could be improved by means of metal doping, control of the structure morphology, coupled with a facile preparation process.

A number of researchers attempted various methods to simplify the preparation process of the gas sensors. For example, Su et al. [33] prepared Pt-PPy thin film by layer-by-layer (LBL) self-assembly method for H_2_ gas sensing. Other methods are utilized as well to enhance gas response including ultrasonic spray pyrolysis [4], screen printing [18,19,22], RF magnetron sputtering [27], electro-spinning [22], chemical vapor deposition [25], electrothermal [29], co-sputtering [17], and matrix assisted pulsed laser evaporation [34]. Drop-coating is also a common method utilized to prepare gas sensors using powder materials [1,2,13,31,32,33,35,36]. Even though these strategies were successful in addressing particular issues, most of them suffer from either high operating temperatures or complex manufacturing processes. 

As for sensors for H_2_ detection, several studies have also been reported including methods based on catalytic, thermal conductivity, electrochemical, resistance, work function, mechanical, and optical and acoustic [37,38,39]. In recent years, solid electrolytes with proton conductivity [40] have attracted increasing attention of electrochemical devices developers. These materials can be implemented in different medium- and high-temperature electrochemical devices, including fuel cells, electrolysis cells for hydrogen production and hydrogen sensors [41,42]. Additionally, Pd nanogap-based sensors on flexible substrates were reported to have some advantages for electrical H_2_ detection, such as a high sensitivity and selectivity to H_2_ at room temperature [43,44]. Metal oxide gas sensors have been widely used for hydrogen gas sensor applications because of their high sensitivity, fast response, long lifespan and feasibility to be miniaturized for use in the microelectronic devices [45,46]. Moreover, the morphologies and structures of metal oxides may play important roles in the sensor characteristics [47,48,49,50,51]. For example, a pure and doped WO_3_ nanoparticle, forming nanoporous layers as testing material was used for improving chemical selectivity and sensitivity of semiconductor gas sensors consisting of nanostructured thin films [47]. Especially, TiO_2_ tubular structures prepared by anodization have shown significant hydrogen sensing properties [48,49]. Paulose et al. [50] reported remarkable hydrogen sensing properties of TiO_2_ nanotube arrays prepared by an anodization of Ti foil in an electrolyte containing fluorine ion. The dramatic change in resistance is believed to be due to the highly active surface states on the nanoscale walls of the tubes, high surface area of the nanotube architecture, and the well-ordered geometry allowing for hydrogen-sensitive tube-to-tube electrical connections. Moon et al. [51] also reported a highly sensitive hydrogen sensor consisting of Pd-decorated TiO_2_ tubular structures prepared by anodization with patterned electrodes on SiO_2_/Si substrate. These works showed that the hydrogen sensing properties of TiO_2_ sensors were somewhat determined by their nanostructures. However, little attention was paid to the photo-assisted sensing properties of TiO_2_ films with different nanostructures. 

In this present work, we have compared the photo-assisted hydrogen sensing properties of two kinds of TiO_2_ films, one prepared by an in-situ method and the other a dip-coating method. Hydrogen sensing measurements were performed under N_2_ and synthetic air. The in-situ TiO_2_ film sensor showed a better hydrogen sensing property than the dip-coating TiO_2_ film sensor.

## 2. Experiments

### 2.1. Fabrication of the Gas Sensor

**Scheme 1:**
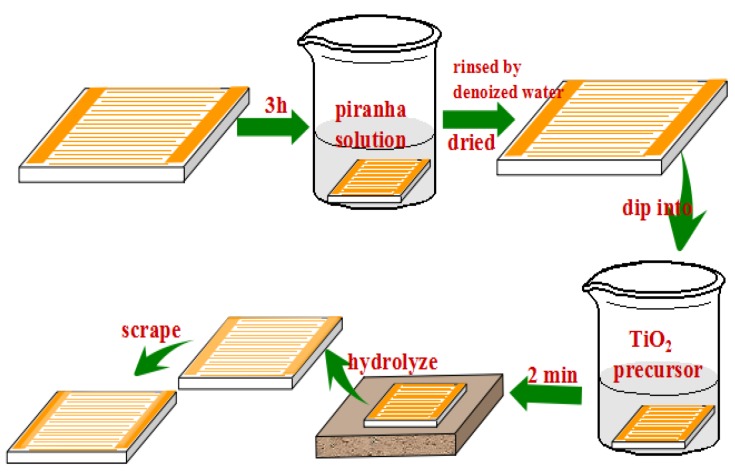
Schematic diagram of the preparation process for TiO_2_ gas sensor sample.

The TiO_2_ gas sensor was prepared by the in-situ method showed in Scheme 1. An interdigitated Au electrode (15 mm × 10 mm, the gap size of 0.15 mm) deposited on the alumina (α-Al_2_O_3_) substrate was used as the substrate for the gas sensor. The electrode was first pretreated subsequently with acetone, ethanol, and deionized water, and then treated with piranha solution (30% H_2_O_2_:98% H_2_SO_4_ equivalent to 3:7 *v/v*) for 3 h in order to increase the hydrophilous character of the electrode surface [29]. The electrode was rinsed several times with deionized water and dried in the oven at 80 °C. In the second step, the clean electrode was dipped into a precursor solution containing a mixture of titanium isopropoxide and ethanol (1:10 *v/v*) for 2 min. Then, the electrode was put on a smooth table to rest and be hydrolyzed naturally through the air humidity. In the final step, the electrode was calcinated at 500 °C for 2 h at a heating rate of 1 °C·min^−1^, and then let to cool down to room temperature. The obtained TiO_2_ gas sensor is labeled as TiO_2_-I. 

For characterization and testing purposes, a TiO_2_ powder sample labeled as TiO_2_ (in-situ) was prepared using the above precursor solution, then exposed to air and subsequent hydrolization and calcination processes.

For comparative purposes, another TiO_2_ sensor device was fabricated by the drop-coating method reported previously [31]. Precisely, A 30 mg of commercial TiO_2_ powder (99.8 wt% anatase, 5–10 nm, Aladdin Chemistry Co. Ltd., Shanghai, China) was dispersed in 1 mL terpineol (Chengjie Chemistry Co. Ltd., Shanghai , China) solvent to obtain a TiO_2_ suspension. The interdigitated Au electrode, subsequently pretreated by ultrasonic treatment in ethanol (99.7% purity) and deionized water then dried in the oven, was treated with 50 μL of the obtained TiO_2_ suspension by dropping the suspension onto the clean electrode surface. The coated electrode coated was then dried at 100 °C for 1 h and calcinated in air at 350 °C for 3 h (heating rate 2 °C·min^−1^). The electrode was left to cool down to room temperature, and the resulting sensor device was labeled as TiO_2_-D. It is worth noting that the corresponding TiO_2_ powder sample was labeled TiO_2_ (commercial).

### 2.2. Characterization

The phase and crystal structure of TiO_2_ powder samples were characterized by X-ray diffraction (XRD, D8 Advance, Brucker, Karlsruhe, Germany) having Cu Kα radiation. UV-vis diffuse reflectance spectra (UV-vis DRS) of TiO_2_ sensor film samples were recorded with UV-vis spectrophotometer (Cary-500, Varian Co., Guangzhou, China). Surface morphology of TiO_2_ sensor (film) samples was performed with Scanning Electron Microscopy (SEM) (Hitachi S4800, Hitachi, Tokyo, Japan).

### 2.3. Photoelectrochemical Measurement

The electrochemical properties of the samples were performed with an electrochemical analyzer having a three-electrode configuration. A fluorine-doped tin oxide (FTO) conductive glass coated with the material film was used as a working electrode, Pt wire as a counter-electrode, and Ag/AgCl (in saturated KCl (aq)) as a reference electrode. An aqueous solution of Na_2_SO_4_ (0.02 M) was used as an electrolyte. The working electrodes were prepared by drop-coating, where the FTO conductive glass electrodes were first washed in an ultrasonic bath with ethanol, deionized water, and then dried at 80 °C for 2 h. A 5 mg powder sample of TiO_2_ was added to 1 mL absolute DMF to make a slurry, and the suspension was then dispersed in an ultrasonic bath for over 3 h. The resulting slurry suspension was injected uniformly onto the conductive surface of 2.5 cm × 1.0 cm FTO glass electrode with the cell size of 5 mm × 5 mm. To obtain conductive working electrodes, the non-conductive nail polish was brushed coating on the conductive surface of FTO glass. The coated FTO glass electrode was then dried at room temperature for about 12 h. The transient photocurrent response for TiO_2_ stacks in the air was recorded on an electrochemical analyzer (Epsilon, BAS, USA) at the operation voltage of 0.5 V with point light as a light source. The electrochemical impedance spectroscopy (EIS) measurements were performed in a three-electrode cell and measurements recorded with Epsilon.

### 2.4. Testing of the Gas Sensors

The gas sensing properties of the films were investigated in a chamber made of stainless steel having a total volume of 100 mL. The response of the sensor films to gas was measured by aJF02Egas sensing test system (GuiYanJinFeng Tech. Co. Ltd., Kunming, China) at controlled voltage of 8.5 V. During the tests, the irradiation source was emitted by four UV lamps (4 W, Philips TL/05, PHILIPS, Poland) having wavelength set at 365 nm and the estimate total light intensity on the sensor surface was about 7.3 mW·cm^−2^. The light distance between diode and the sensor sample was about 4.5 cm. During this process, high purity N_2_ or air was used as background atmosphere. The air atmosphere was a synthetic air consisted of 20.5 vol% O_2_ and 79.5 vol% N_2_. Prior to any measurement, the sensor films were maintained at temperatures of 200 °C for 1 h in the corresponding background atmosphere (N_2_ or the synthetic air) and then cooled down to room temperature. Afterward, a probe gas H_2_ diluted in gas high purity N_2_ was introduced into the chamber at a flow rate of 250 mL·min^−1^ instead of the background atmosphere. Since the gas sensitivity of two samples to H_2_ was weak (the response limit of H_2_ concentration was about 5000 ppm, the detailed testing curves are not shown here), the H_2_ concentration was controlled at 20,000 ppm so as to clearly identify the differences between two samples. The response of the sensors to H_2_ was reflected in the variation of the impedance spectroscopy data induced by changes in surface conductivity (the highest detection limit is 10^6^ KΩ). The gas sensing response (Sr) of the sensor was defined as: Sr = R_0_/R_gas_, where R_0_ and R_gas_ were the impedance values measured in the background atmosphere and in the testing gas [31], respectively. Herein, the value of Sr may reflect the gas sensing performance of the sensor to the probe gas. 

Moreover, the thermal responses to H_2_ of the sensor films were also performed at the same condition under no UV irradiation. Here, the film samples were maintained at 400 °C.

Since the metal oxide based gas sensors are sensitive to humidity, the relative humidity during the gas testing was carried out by a thermo-hygrometer, and the relative humidity during the testing process kept at about 74%. 

## 3. Results and Discussion

### 3.1. Properties of TiO_2_ Sensor Films

**Figure 1:**
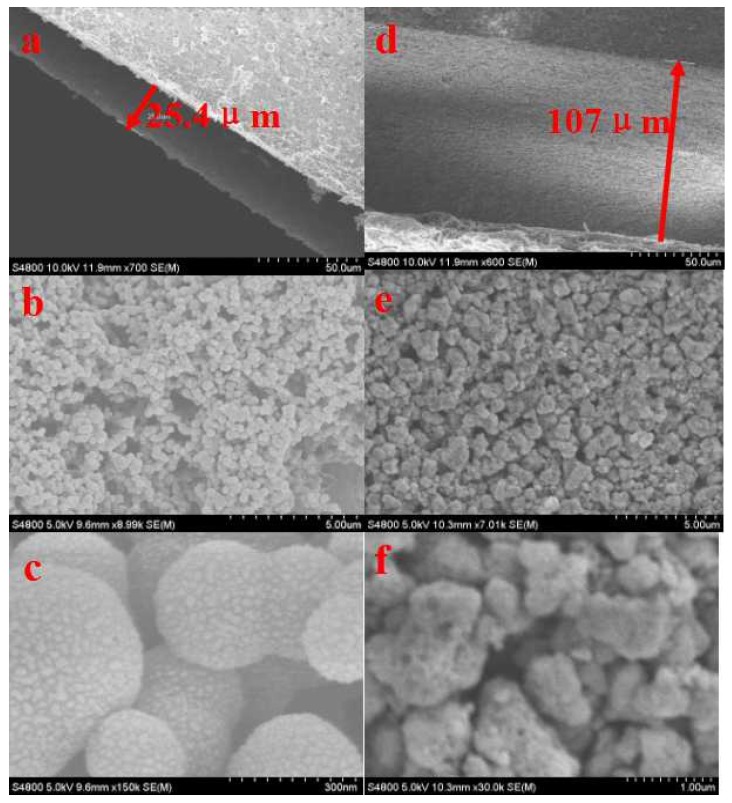
SEM images of TiO_2_ gas sensor samples: (**a**–**c**) the in-situ prepared TiO_2_ sensor sample (TiO_2_-I); and (**d**–**f**) the drop-coating prepared TiO_2_ sensor sample (TiO_2_-D).

Figure 1 shows the surface morphology of the TiO_2_ sensor films. For the TiO_2_-I, a film with 25.4 μm thickness was deposited on the electrode surface (Figure 1a). As shown in Figure 1b,c, the TiO_2_ film was mainly composed of overlaid TiO_2_ microspheres with sizes ranging from 200 to 300 nm. Moreover, a porous structure could be seen among the adjacent microspheres that may contribute to an increase in the surface area that eventually would be conductive to adsorb more of the probe gas molecules [52]. In the TiO_2_-D sample, the TiO_2_ film loaded on the electrode substrate was mainly composed of nanoparticles stacked by a large of random granular (Figure 1d). The TiO_2_-D sensor film was about 109 μm, which was thicker than TiO_2_-I gas sensor. Figure 1e,f illustrates that the TiO_2_ nanoparticles underwent an aggregation after the calcination process of the substrates. TiO_2_ (commercial) was 5–10 nm before calcined, it may aggregate on the substrate result in the TiO_2_ nanoparticles were not uniform in size, which bank up the thick film. Considering that the morphology of the films may play an important role in the sensor characteristics including the sensitivity [15,21,25,26], the morphological differences between TiO_2_-I and TiO_2_-D based samples may be one of the important reasons leading to differences in sensitivities between the sensors.

**Figure 2:**
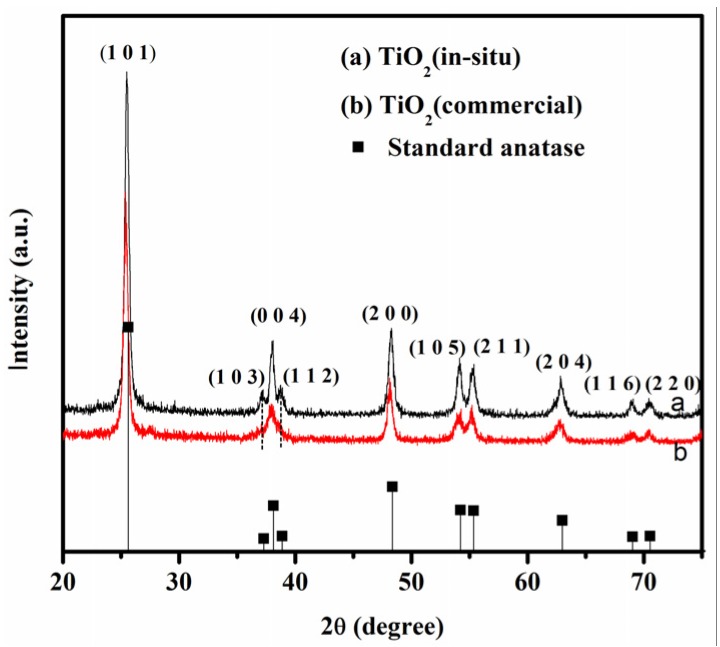
The XRD spectra of (**a**) TiO_2_ (in-situ) sample; and (**b**) TiO_2_ (commercial) samples.

Since the location of the XRD peaks of α-Al_2_O_3_ [53] and Au [20] are very close to those of anatase TiO_2_, the XRD patterns of the two TiO_2_ powder samples without electrode substrate (containing α-Al_2_O_3_ and Au components) were recorded. As seen in Figure 2, Both TiO_2_ (in-situ) and TiO_2_ (commercial) showed a number of characteristic diffraction peaks located at 2θ = 25.37°, 37.8°, 48.1°, 54.0°, 55.1°, 62.8°, 68.8° and 72.3°, which could be attributed, respectively, to (101), (004), (200), (105), (211), (204), (116) and (220) crystal planes of anatase TiO_2_ (JCPDS card 75-1537). In addition, two small peaks at 36.9° and 38.6° in TiO_2_ (in-situ) could be assigned to (103) and (112) crystal planes of anatase TiO_2_, respectively. Note that the two peaks were too weak to be observed in TiO_2_ (commercial). Moreover, the two TiO_2_ samples owned the similar crystal sizes of anatase structure (19.6 nm of TiO_2_ (in-situ) vs. 18.2 nm of TiO_2_ (commercial)) according to the Debye–Scherrer formula. The similarity between the XRD features of both samples indicates that the TiO_2_ (in-situ) was formed in its pure anatase crystalline structure similar to the TiO_2_ (commercial) sample. 

**Figure 3:**
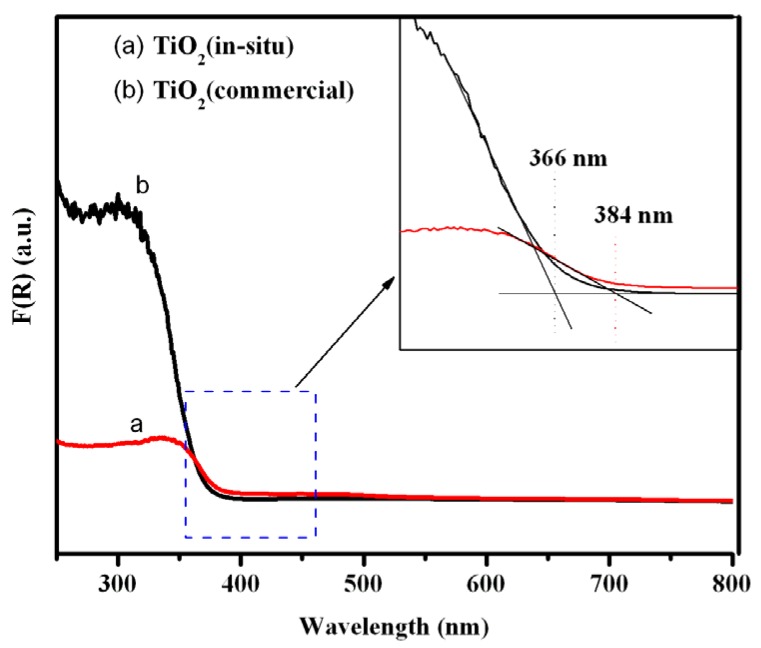
UV-vis diffuse reflectance spectra (DRS) of: (**a**) TiO_2_ (in-situ) sample; and (**b**) TiO_2_ (commercial) samples.

Figure 3 shows the UV-vis diffuse reflectance spectra of TiO_2_-I and TiO_2_-D samples. Both samples represent the TiO_2_ intrinsic absorption band in the ultraviolet region. If compared to TiO_2_-D, the TiO_2_-I exhibits weak absorption intensity, it may due to the TiO_2_-I film was thinner than the TiO_2_-D. While a red shift in the basal absorption region from round 366 nm to 380 nm was observed, it could be attributed to the microsphere structure of the TiO_2_-I sample observed in Figure 1 [54,55]. This indicates that TiO_2_-I has a wider absorption UV-light region than TiO_2_-D, which may result in a stronger photo-response during gas sensing testing [56].

### 3.2. Performance of TiO_2_ Gas Sensor

**Figure 4:**
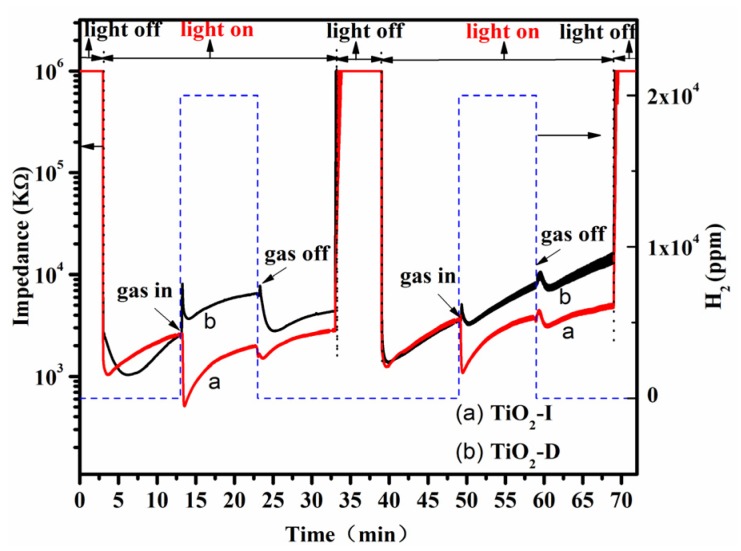
Gas sensing processes to H_2_ under UV light irradiation at room temperature in N_2_ atmosphere over two different samples: (**a**) the in-situ prepared TiO_2_ sensor sample (TiO_2_-I); and (**b**) the drop-coating prepared TiO_2_ sensor sample (TiO_2_-D). The solid curves denote the impedance module of samples as function of time, and the dotted line denotes the concentration of H_2_ during the testing process.

Figure 4 shows the photo-assisted gas sensing performance of TiO_2_-I and TiO_2_-D samples to H_2_ in N_2_ atmospheres at room temperature under the UV irradiation. It could be seen that the impedance of both TiO_2_-I and TiO_2_-D decreased rapidly upon the introduction of the UV light under the pure N_2_ atmosphere, then increased gradually during the following time of the irradiation, which is in agreement with our previous report [31]. The reason for this may have to do with the UV light that could excite TiO_2_ to produce photo-generated electrons. In turn, this resulted in an enhancement in the surface electron density of TiO_2_ nanoparticles and a decrease in the impedance of the sample. However, UV light might cause the change in the surface state of TiO_2_ (in fact, the process of UV irradiating TiO_2_ can be regarded as a process of TiO_2_ reduced by UV light), the generation of electrons, the recombination of electrons with holes, the capture of electrons by defects or surface hydroxyls, and the transfer of electrons will make some changes with the duration of introducing UV light, especially under the bias voltage. Therefore, the impedance of TiO_2_ sensor sample increased slowly with the duration of UV irradiation [57]. With the introduction of H_2_, the impedance of TiO_2_-I sample decreased rapidly but that of TiO_2_-D sample increases. The latter indicates that the adsorbed H_2_ could offer electrons to TiO_2_-I but accept electrons from the TiO_2_-D sample [57]. Moreover, TiO_2_-I exhibited a higher response to H_2_ than TiO_2_-D (Table 1). As can be seen in Table 1, the value of the gas sensing response of the TiO_2_-I in first circle was about 4.856, while TiO_2_-D was just 0.738. This illustrates that the gas sensing performance of TiO_2_-I to H_2_ may be superior to TiO_2_-D in N_2_ atmospheres at room temperature under the UV irradiation. By removing H_2_, the impedance of TiO_2_-D decreases under the UV irradiation, confirming that the adsorbed H_2_ could accept electrons from TiO_2_-D. On the other hand, the TiO_2_-I sample also exhibited a slight decrease in impedance with the removal of H_2_, indicating that H_2_ could also accept electrons from TiO_2_-I during the late stages of the process. After H_2_ stream was cut off, the impedance of TiO_2_-I increased slowly with the duration of UV irradiation in N_2_ atmosphere, which present the same trend as that process prior to introducing H_2_. Note that TiO_2_-I also exhibited a shorter response time and a shorter recovery time as compared to TiO_2_-D (seen in Table 1).

Additionally, TiO_2_-I showed a change in impedance (gas sensitivity) in the second cycle similar to the one observed during the first cycle, but the TiO_2_-D sample showed a different response from the one seen during the first cycle (changes from a negative to a positive response). Furthermore, the gas sensing response value of TiO_2_-I was also bigger than TiO_2_-D. This means that the TiO_2_-I sample has a more stable sensitivity to H_2_ in N_2_ atmosphere under the UV irradiation than the TiO_2_-D.

**Figure 5:**
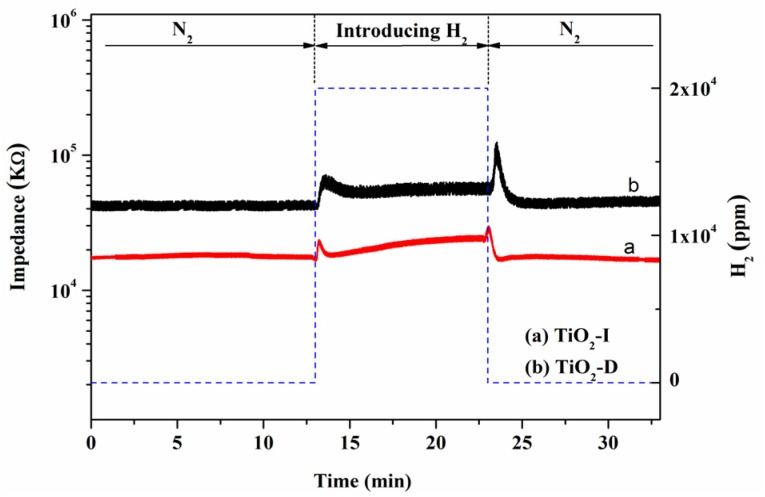
Gas sensing process to H_2_ in N_2_ atmosphere at 400 °C without UV light over: (**a**) TiO_2_-I samples; and (**b**) TiO_2_-D samples. The solid curves denote the impedance module of samples as function of time, and the dotted line denotes the concentration of H_2_ during the testing process.

Moreover, the thermal sensing properties of two samples were also tested. As seen in Figure 5, both the TiO_2_-I and TiO_2_-D samples exhibited a weak response to H_2_ in N_2_ atmosphere with the applied voltage of 8.5 V under no UV irradiation at 400 °C. Moreover, the response value of each sample was much weaker than the respective response value at the same condition under UV irradiation (see Figure 4), indicating that the photo-assisted sensitivity of each sample may be stronger than the respective thermal (chemical) sensitivity at 400 °C. Moreover, the thermal sensitivity of TiO_2_-I at 400 °C was also stronger than that at 250 °C, 300 °C and 350 °C (see Figure S1). Note that the thermal response values of two samples were more stable than the photo-assisted response values. However, the thermal sensitivities of two samples towards H_2_ are lower than other H_2_ gas sensors using TiO_2_ nanostructures in the literatures, e.g., TiO_2_ nanotube sensors [51]. Considering the promotion effect of UV light on the H_2_ sensitivities of the two above TiO_2_ samples in this work, we think that the gas sensitivities of the TiO_2_ nanotube sensors may be further enhanced by introducing UV light.

**Figure 6:**
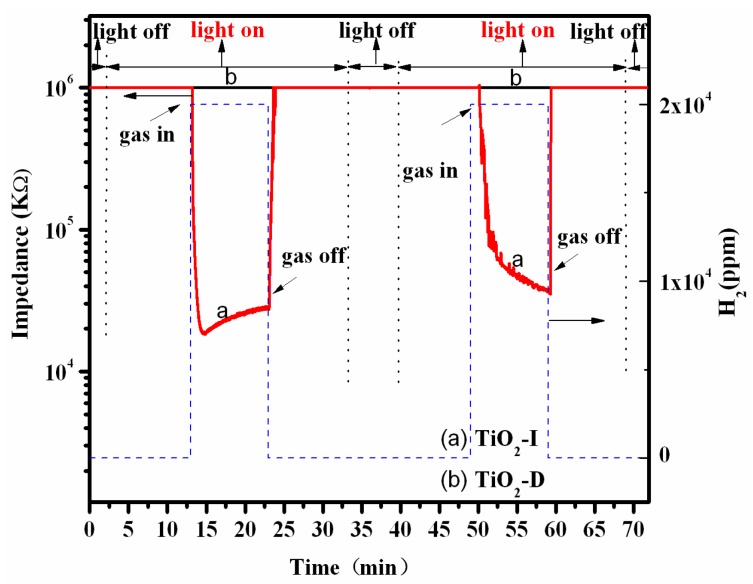
Gas sensing processes to H_2_ in air atmosphere under UV light irradiation at room temperature over: (**a**) TiO_2_-I samples; and (**b**) TiO_2_-D samples. The solid curves denote the impedance module of samples as function of time, and the dotted line denotes the concentration of H_2_ during the testing process.

Furthermore, the photo-assisted gas sensitivities to H_2_ of both TiO_2_-I and TiO_2_-D samples were also performed in the air atmosphere instead of N_2_. As displayed in Figure 6, both TiO_2_-I and TiO_2_-D showed no response in the air atmosphere when irradiated under the UV irradiation. Upon the introduction of H_2_, TiO_2_-D still showed no response as there is no decrease in the impedance. However, the TiO_2_-I sample exhibited an obvious response to H_2_ as a rapid decrease in the impedance could be noticed. Because the base impedance value reached the detection limit, the response value was very large respect to Table 1. With the removal of H_2_, the impedance of TiO_2_-I rapidly increased again. Moreover, the TiO_2_-I sample still illustrated a response to H_2_ in the air atmosphere under the UV irradiation during the second cycle. This above result indicated that the TiO_2_-I sensor could be reused to gas sensing response to H_2_, while TiO_2_-D could not. However, the response transition of TiO_2_-I sample in the second cycle was different from that in the first cycle, i.e., the impedance of TiO_2_-I sample increased slowly during the process of introducing H_2_ in the first cycle, but decreased slowly in the second cycle. 

In other words, if compared to the drop-coating prepared TiO_2_ sensor (TiO_2_-D), the in-situ prepared TiO_2_ sensor (TiO_2_-I) exhibited a superior gas sensitivity towards H_2_ under the UV irradiation at room temperature in both N_2_ and synthetic air atmospheres. The uniform TiO_2_ microspheres present in the TiO_2_-I sample might play a significant role in the superior efficiency of the photo-assisted gas sensors. This latter may be attributed to the microsphere structure that could promote the generation and transfer of the photo-generated electrons under the UV radiation [55]. Although the presence of O_2_ could weaken the photo-assisted response to H_2_ in TiO_2_ due to the adsorbed O_2_ molecules that could accept the photo-generated electrons from TiO_2_ [31], the TiO_2_-I sample still present an effective response to H_2_ in the air atmosphere (Figure 6). This indicates that the presence of H_2_ may suppress the ability of O_2_ in accepting the generated electrons from TiO_2_ over TiO_2_-I. As for the different response transitions of TiO_2_-I towards H_2_ between the first cycle and the second cycle (see Figure 6), it may be attributed to the competitive adsorption of H_2_ and O_2_ at different adsorption sites of TiO_2_ induced by the order of adsorbing two gases. A detailed explanation needs to be further investigated. By contrast, TiO_2_-D shows practically no response to H_2_ in the air atmosphere under the UV irradiation (Figure 6), indicating that the presence of H_2_ does not suppress the behavior of the adsorbed O_2_ to accept electrons from TiO_2_. This positive result of TiO_2_-I sample towards H_2_ in air atmosphere shows that the change of structure or morphology of TiO_2_ might cause a transformation in its gas sensing property. Of course, the thickness difference between TiO_2_-I and TiO_2_-D (see Figure 2) may also cause the change in H_2_ sensing property of two samples according to the report of Sakai et al. [58]. However, it was difficult to obtain the same thickness of TiO_2_ film for TiO_2_-I and TiO_2_-D samples due to the different preparing method. Moreover, the different nanostructures and morphologies existed in two samples also make it difficult to compare the changes of sensitivity induced by TiO_2_ film thickness. The detailed reason needs to be investigated in the further work.

### 3.3. Photoelectrochemical Properties of TiO_2_

Yamazoe et al. [59] have reported that the gas sensing performance of a conductometric semiconductor sensor is mainly determined by the following three key factors: (a) a base oxide with high mobility of conduction electrons and satisfactory stability; (b) a foreign receptor which enhances surface reactions or adsorption of target gas; and (c) the fabrication of a highly porous, thin sensing body of oxide. This indicates that the grain size of oxide will exert an effect on the gas sensitivity by adsorbing target gas at the surface of each oxide grain and the electron transfer between each grain boundary. Considering that the structure and grain size of TiO_2_-I were different from those of TiO_2_-D, the electron transfer behaviors of two samples under UV irradiation were compared by the photoelectrochemical measurements.

**Figure 7:**
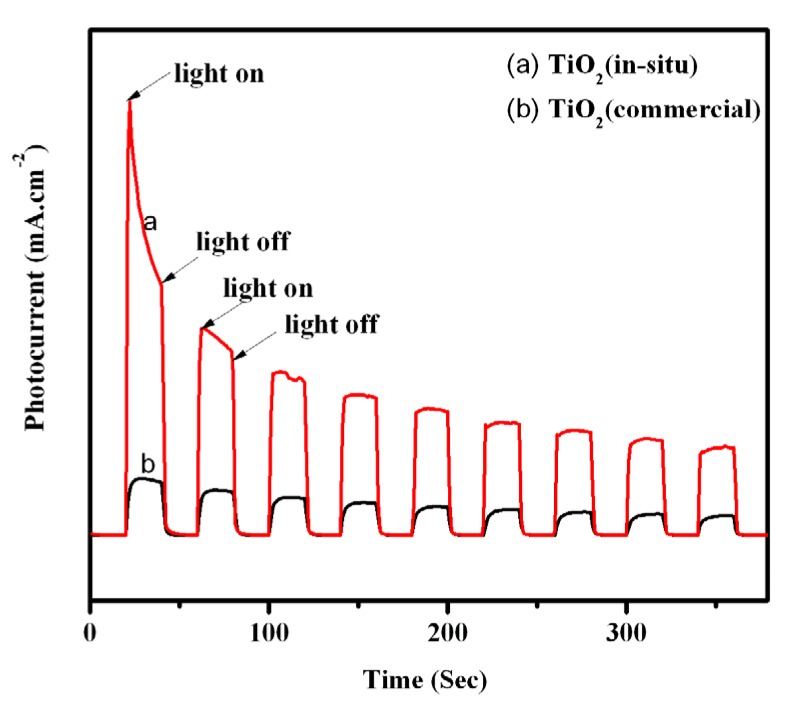
Transient photocurrent responses of: (**a**) TiO_2_ (in-situ) samples; and (**b**) TiO_2_ (commercial) samples in 0.02 M Na_2_SO_4_ aqueous solution under UV light irradiation.

Figure 7 illustrates the photocurrent results of the TiO_2_ (in-situ) and TiO_2_ (commercial) electrodes upon ON and OFF time of the UV irradiation. Both samples exhibited an increase in the photocurrent intensities under the UV irradiation and a decrease upon removal of the UV light. This indicates that not only extra electrons could be produced over TiO_2_ under UV irradiation, but also the photo-generated electrons could undergo a transfer from the TiO_2_ surface to the indium tin oxide substrates in both electrodes. Obviously, it could be observed that the photocurrent of the TiO_2_ (in-situ) electrode is much larger than the one obtained with the TiO_2_ (commercial) electrode. With the irradiation prolonged, more and more photo-generated electrons and holes recombined or be captured on TiO_2_ surface due to the reduction of TiO_2_ by UV light [57], which resulted in the photocurrent decay. Note that the photocurrent value of TiO_2_ (in-situ) is larger than that of TiO_2_ (commercial), indicating that the recombination rate of TiO_2_ (in-situ) is lower than that of TiO_2_ (commercial). 

This above result demonstrates that the TiO_2_ (in-situ) electrode was more efficient in the generation, separation, and transfer of the photo-generated electron–hole pairs [47,54,60] when compared with TiO_2_ (commercial). That is to say, the one-step method prepared sensor sample might form a strong interaction between TiO_2_ nanoparticle and electrode substrate or among TiO_2_ nanoparticles, which would be propitious to the electron transfer and the charge separation. This could be the reason of why the in-situ prepared TiO_2_ sensor (TiO_2_-I) displays superior gas sensing performance towards H_2_ than the drop-coating TiO_2_ sensor (TiO_2_-D). In addition, the different thickness and roughness of the gas sensing layer between two samples might also somewhat lead to the observed differences. A more description needs to be further investigated. 

**Figure 8:**
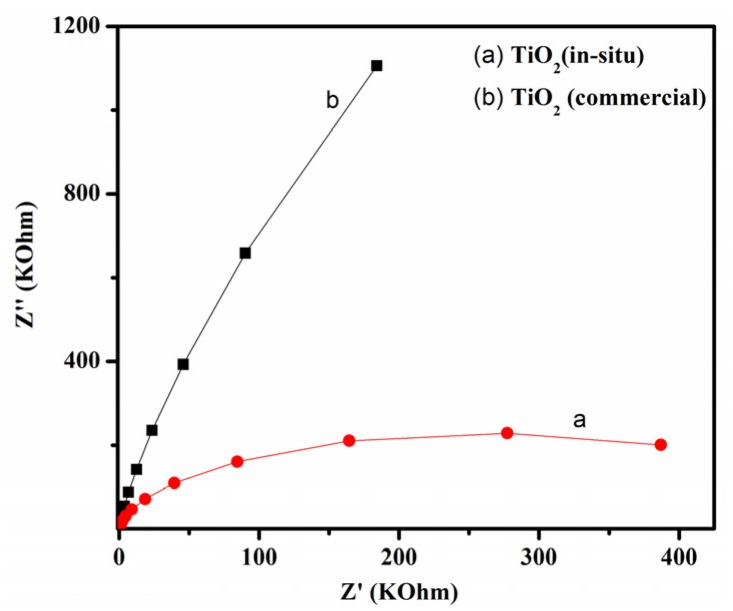
EIS Nyquist plots of: (**a**) TiO_2_ (in-situ) samples; and (**b**) TiO_2_ (commercial) samples. The amplitude of the sinusoidal wave was set at 10mV and the frequency varied from 100 kHz to 0.05 Hz. The Z' and Z'' correspond to the real part and the imaginary part of the impedance, as the horizontal and vertical axis in the impedance spectra, respectively.

The above results of electron transfer were further confirmed by EIS measurements, an effective tool that provides useful information on impedance changes in modified electrode surfaces [61,62]. In general, a smaller semi-circle in the EIS Nyquist plots indicates the smaller resistance at the material interface, and thus more effective interfacial charge transfer at the electrode interface [62,63]. As shown in Figure 8, the size of the semi-circle radius on the Nyquist plots of TiO_2_ (in-situ) was smaller than the one obtained with TiO_2_ (commercial) electrode. This reveals that the TiO_2_ (in-situ) electrode achieves a better surface electron transfer. In fact, Ding et al. [64] have reported that adding one layer of TiO_2_ nano-crystalline as a linking bridge between TiO_2_ sub-microspheres layer and substrates could promote a better electron transfer through a high specific surface area and effective light scattering. Accordingly, it is possible that the unique nanostructure of TiO_2_ films formed by uniform TiO_2_ microspheres in the in-situ prepared TiO_2_ sensor could benefit the electron transfer and charge separation between TiO_2_ and substrates or among TiO_2_ nanoparticles, resulting in the higher photo-assisted gas sensitivity. This result also shows that a new photo-electrochemical measurement may be used to investigate the electron transfer behavior in TiO_2_ or other semiconductor gas sensors.

Of course, this prepared TiO_2_ sensor sample cannot yet be applied for detecting H_2_. It still needs to be further improved in some cases, especially its sensing stability. However, the positive result of response to H_2_ in air showed that this work might offer an approach to improve the photo-assisted sensing properties of TiO_2_ and other semi-conductors by changing their structures and morphologies. We also hope that this photo-assisted gas sensing property will occur on other TiO_2_ nanostructure sensors with the better thermal gas sensitivity, such as TiO_2_ nanotube sensor. The related work will be processed in the near future. 

## 4. Conclusions

From the present investigation the following conclusions can be drawn:

- A TiO_2_-I film sensor formed by uniform TiO_2_ microspheres, fabricated by a facile in-situ method, which exhibited an effective sensitivity towards H_2_ in both N_2_ atmosphere and synthetic air at the room temperature under UV irradiation.
- A TiO_2_-D film sensor, prepared by drop-coating method, exhibited an apparent sensitivity towards H_2_ only in N_2_ atmosphere but not in synthetic air under UV irradiation.
- The better photo-assisted sensitivity of TiO_2_-I could be attributed to the more effective charge transfer at TiO_2_ interface, which can be evaluated by the photoelectrochemical measurement.
- The effective charge transfer behaviors in TiO_2_-I could be originated from the uniform TiO_2_ nanosphere structure.
- This study shows that changing the structure and morphology of film sensor (e.g., preparing a uniform nanostructure) maybe improve the photo-assisted sensing properties of TiO_2_ and other semi-conductor sensors by enhancing electron transfer efficiency.

## Figures and Tables

**Table 1:** Photo-assisted gas sensing response to H_2_ in N_2_ atmosphere under the UV irradiation over (**a**) the in-situ prepared TiO_2_ sensor sample (TiO_2_-I) and (**b**) the drop-coating prepared TiO_2_ sensor sample (TiO_2_-D) in Figure 4. Since the impedance values under UV irradiation seemed not to be stable, the R_0_ was assigned to the last impedance value prior to introducing H_2_, while the R_gas_ was assigned to the lowest value after introducing H_2_.

Samples	R_0_ (kΩ)	R_gas_ (kΩ)	Sr = R_0_/R_gas_	Response Time (s)	Recovery Time (s)
TiO_2_-I (First cycle)	2564	528	4.856	18	56
TiO_2_-I (Second cycle)	3636	1100	3.305	18	113
TiO_2_-D (First cycle)	2698	3658	0.738	56	90
TiO_2_-D (Second cycle)	3403	3147	1.081	56	102

## Supplementary Materials

The following are available online at http://www.mdpi.com/1424-8220/16/8/1249/s1, Figure S1: Gas sensing process to H_2_ in N_2_ atmosphere at different temperatures without UV light over TiO_2_-I sample. The solid curves denote the impedance module of samples as function of time, and the dotted line denotes the concentration of H_2_ during the testing process. However, the testing process at 450 °C could not be performed due to the temperature limit of the chamber.

## References

[1] Usha S.P., Mishra S.K., Gupta B.D. (2015). Fiber optic hydrogen sulfide gas sensors utilizing ZnO thin film/ZnO nanoparticles: A comparison of surface plasmon resonance and lossy mode resonance. Sens. Actuators B Chem..

[2] Cuong N.D., Khieu D.Q., Hoa T.T., Quang D.T., Viet P.H., Lam T.D., Hoa N.D., Hieu N.V. (2015). Facile synthesis of α-Fe_2_O_3_ nanoparticles for high-performance CO gas sensor. Mater. Res. Bull..

[3] Blais F. (2004). Review of 20 years of range sensor development. J. Electron. Imaging.

[4] VinothKumar J., Maldonado A., Olvera M. (2015). A simple and cost-effective zinc oxide thin film sensor for propane gas detection. Mater. Lett..

[5] Grieshaber D., MacKenzie R., Vörös J., Reimhult E. (2008). Electrochemical biosensors—Sensor principles and architectures. Sensors.

[6] Homola J. (2008). Surface plasmon resonance sensors for detection of chemical and biological species. Chem. Rev..

[7] Fujishima A., Honda K. (1972). Electrochemical Photolysis of Water at a Semiconductor Electrode. Nature.

[8] Grätzel M. (2001). Photoelectrochemical cells. Nature.

[9] Konstantinou I.K., Albanis T.A. (2004). TiO_2_-assisted photocatalytic degradation of azo dyes in aqueous solution: Kinetic and mechanistic investigations: A review. Appl. Catal. B Environ..

[10] Ni M., Leung M.K.H., Leung D.Y.C., Sumathy K. (2007). A review and recent developments in photocatalytic water-splitting using TiO_2_ for hydrogen production. Renew. Sustain. Energy Rev..

[11] Millis A., Hunte S.L. (1997). An overview of semiconductor photocatalysis. J. Photochem. Photobiol. A Chem..

[12] Hazra A., Das S., Kanungo J., Sarkar C.K., Basu S. (2013). Studies on a resistive gas sensor based on sol–gel grown nanocrystalline p-TiO_2_ thin film for fast hydrogen detection. Sens. Actuators B Chem..

[13] Lim S.K., Hong S.H., Hwang S.H., Choi W.M., Kim S.H., Park H.W., Jeong M.G. (2015). Synthesis of Al-doped ZnO Nanorods via Microemulsion Method and Their Application as a CO Gas Sensor. J. Mater. Sci. Tech..

[14] Bai J., Zhou B. (2014). Titanium dioxide nanomaterials for sensor applications. Chem. Rev..

[15] Moon J., Park J.A., Lee S.J., Zyung T., Kim I.D. (2010). Pd-doped TiO_2_ nanofiber networks for gas sensor applications. Sens. Actuators B Chem..

[16] Sharma K.R., Bhatnagar M.C., Sharma G.L. (1997). Mechanism of highly sensitive and fast response Cr doped TiO_2_ oxygen gas sensor. Sens. Actuators B Chem..

[17] Comini E., Sberveglieri G., Ferroni M., Guidi V., Martinelli G. (2003). Response to ethanol of thin films based on Mo and Ti oxides deposited by sputtering. Sens. Actuators B Chem..

[18] Ruiz A.M., Cornet A., Morante J.R. (2004). Study of La and Cu influence on the growth inhibition and phase transformation of nano-TiO_2_ used for gas sensors. Sens. Actuators B Chem..

[19] Bonini N., Carotta M.C., Chiorino A., Guidi V., Malagù C., Martinelli G., Paglialonga L., Sacerdoti M. (2000). Doping of a nanostructured titania thick film: Structural and electrical investigations. Sens. Actuators B Chem..

[20] Zhu Z., Chang J.L., Wu R.J. (2015). Fast ozone detection by using a core–shell Au@TiO_2_ sensor at room temperature. Sens. Actuators B Chem..

[21] Plecenik T., Moško M., Haidry A.A., Ďurina P., Truchlý M., Grančič B., Gregor M., Roch T., Satrapinskyy L., Mošková A. (2015). Fast highly-sensitive room-temperature semiconductor gas sensor based on the nanoscale Pt–TiO_2_–Pt sandwich. Sens. Actuators B Chem..

[22] Kobayashi H., Kishimoto K., Nakato Y., Tsubomura H. (1993). Mechanism of hydrogen sensing by Pd/TiO_2_ Schottky diodes. Sens. Actuators B Chem..

[23] Walton R.M., Liu H., Gland J.L., Schwank J.W. (1997). Resistance measurements of platinum-titania thin film gas detectors in ultra-high vacuum (UHV) and reactive ion etcher (RIE) systems. Sens. Actuators B Chem..

[24] Mor G.K., Varghese O.K., Paulose M., Ong K.G., Grimes C.A. (2006). Fabrication of hydrogen sensors with transparent titanium oxide nanotube-array thin films as sensing elements. Thin Solids Films.

[25] Gao M., Ito A., Goto T. (2015). Preparation of γ-Al_2_O_3_ films by laser chemical vapor deposition. Appl. Surf. Sci..

[26] Sonker R.K., Sabhajeet S.R., Singh S., Yadav B.C. (2015). Synthesis of ZnO nanopetals and its application as NO_2_ gas sensor. Mater. Lett..

[27] Nikfarjam A., Salehifar N. (2015). Improvement in gas-sensing properties of TiO_2_ nanofiber sensor by UV irradiation. Sens. Actuators B Chem..

[28] Gong J., Li Y.H., Chai X.S., Hu Z.S., Deng Y.L. (2010). UV-light-activated ZnO fibers for organic gas sensing at room temperature. J. Phys. Chem. C.

[29] Camagni P., Fanglia G., Galinetto P., Perego C., Samoggia G., Sberveglieri G. (1996). Photosensitivity activation of SnO_2_ thin film gas sensors at room temperature. Sens. Actuators B Chem..

[30] Cao C.L., Hu C.H., Wang X., Wang S.X., Tian Y.S., Zhang H.L. (2011). UV sensor based on TiO_2_ nanorod arrays on FTO thin film. Sens. Actuators B Chem..

[31] Geng Q., He Z.J., Chen X., Dai W.X., Wang X.X. (2013). Gas sensing property of ZnO under visible light irradiation at room temperature. Sens. Actuators B Chem..

[32] Chen H., Liu Y., Xie C.S., Wu J., Zeng D.W., Liao Y.C. (2012). A comparative study on UV light activated porous TiO_2_ and ZnO film sensors for gas sensing at room temperature. Ceram. Int..

[33] Su P.G., Shiu C.C. (2011). Flexible H_2_ sensor fabricated by layer-by-layer self-assembly of thin films of polypyrrole and modified in situ with Pt nanoparticles. Sens. Actuators B Chem..

[34] Rella R., Spadavecchia J., Manera M.G., Capone S., Taurino A., Martino M., Caricato A.P., Tunno T. (2007). Acetone and ethanol solid-state gas sensors based on TiO_2_ nanoparticles thin film deposited by matrix assisted pulsed laser evaporation. Sens. Actuators B Chem..

[35] Kawano T., Chiamori H.C., Suter M., Zhou Q., Sosnowchik B.D., Lin L.W. (2007). An Electrothermal Carbon Nanotube Gas Sensor. Nano Lett..

[36] Vanaraja M, Muthukrishnan K., Boomadevi S., Karn R.K., Singh V., Singh P.K., Pandiyana K. (2016). Dip coated nanostructured ZnO thin film: Synthesis and application. Ceram. Int..

[37] Hübert T., Boon-Brett L., Black G., Banach U. (2011). Hydrogen sensors—A review. Sens. Actuators B Chem..

[38] Fadeyev G., Kalyakin A., Gorbova E., Brouzgou A., Demin A., Volkov A., Tsiakaras P. (2015). A simple and low-cost amperometric sensor for measuring H_2_, CO, and CH4. Sens. Actuators B Chem..

[39] Lee E.B., Hwang I.S., Cha J.H., Lee H.J., Lee W.B., Pak J.J., Lee J.H., Ju B.K. (2011). Micromachined catalytic combustible hydrogen gas sensor. Sens. Actuators B Chem..

[40] Kalyakin A., Fadeyev G., Demin A., Gorbova E., Brouzgou A., Volkov A., Tsiakaras P. (2014). Application of Solid oxide proton-conducting electrolytes for amperometric analysis of hydrogen in H_2_+N_2_+H_2_O gas mixtures. Electrochim. Acta.

[41] Iwahara H., Asakura Y., Katahira K., Tanaka M. (2004). Prospect of hydrogen technology using proton-conducting ceramics. Solids State Ion..

[42] Schwandt C., Fray D.J. (2006). The titanium/hydrogen system as the solid-state reference in high-temperature proton conductor-based hydrogen sensors. J. Appl. Electrochem..

[43] Lee J., Shim W., Lee E., Noh J., Lee W. (2011). Highly mobile palladium thin films on an elastomeric substrate: Nanogap-based hydrogen gas sensors. Angew. Chem. Int. Ed..

[44] Jang B., Lee K., Noh J., Lee W. (2014). Nanogap-based electrical hydrogen sensors fabricated from Pd-PMMA hybrid thin films. Sens. Actuators B Chem..

[45] Dwivedi D., Dwivedi R., Srivastava S.K. (2000). Sensing properties of palladium-gate MOS (Pd-MOS) hydrogen sensor-based on plasma grown silicon dioxide. Sens. Actuators B Chem..

[46] Aroutiounian V. (2007). Metal oxide hydrogen, oxygen and carbon monoxide sensorsfor hydrogen setups and cells. Int. J. Hydrog. Energy.

[47] Heszler P., Ionescu R., Llobet E., Reyes L.F., Smulko J.M., Kish L.B., Granqvist C.G. (2007). On the selectivity of nanostructured semiconductor gas sensors. Phys. Status Solids B.

[48] Mor G.K., Varghese O.K., Paulose M., Shankar K., Grimes C.A. (2006). A review on highly ordered, vertically oriented TiO_2_ nanotube arrays: Fabrication, material properties, and solar energy applications. Sol. Energy Mater. Sol. Cells.

[49] Roy P., Berger S., Schmuki P. (2011). TiO_2_ nanotubes: Synthesis and applications. Angew. Chem. Int. Ed..

[50] Paulose M., Varghese O.K., Mor G.K., Grimes C.A., Ong K.G. (2006). Unprecedented ultra-high hydrogen gas sensitivity in undoped titania nanotubes. Nanotechnology.

[51] Moon J., Hedman H., Kemell M., Tuominen A., Punkkinen R. (2016). Hydrogen sensor of Pd-decorated tubular TiO_2_ layer prepared by anodization with patterned electrodes on SiO_2_/Si substrate. Sens. Actuators B Chem..

[52] Cui C., Qiu Y.W., Zhao J.H., Lu B.Q., Hu H.H., Yang Y.N., Ma N., Xu S., Xu L.B., Li X.Y. (2015). A comparative study on the quantum-dot-sensitized. dye-sensitized and co-sensitized solar cells based on hollow spheres embedded porous TiO_2_ photoanodes. Electrochim. Acta.

[53] Li B., Shao L.L. (2008). The identifying of Al_2_O_3_ and Al(OH)_3_ by XRD. Inorg. Chem. Ind..

[54] Pal S., Laera A.M., Licciulli A., Catalano M., Taurino A. (2014). Biphase TiO_2_ Microspheres with Enhanced Photocatalytic Activity. Ind. Eng. Chem. Res..

[55] Zheng X.Z., Meng S.G., Chen J., Wang J.J., Xian J.J., Shao Y., Fu X.Z., Li D.Z. (2013). Titanium dioxide photonic crystals with enhanced photocatalytic activity: Matching photonic band gaps of TiO_2_ to the absorption peaks of dyes. J. Phys. Chem. C.

[56] Dai J., Yang J., Wang X.H., Zhang L., Li Y.J. (2015). Enhanced visible-light photocatalytic activity for selective oxidation of amines into imines over TiO_2_(B)/anatase mixed-phase nanowires. Appl. Surf. Sci..

[57] Peng X.Y., He Z.J., Yang K., Chen X., Wang X.X., Dai W.X., Fu X.Z. (2016). Correlation between donating or accepting electron behavior of the adsorbed CO or H_2_ and its oxidation over TiO_2_ under ultraviolet light irradiation. Appl. Surf. Sci..

[58] Sakai G., Matsunaga N., Shimanoe K., Yamazoe N. (2001). Theory of gas-diffusion controlled sensitivity for thin film semiconductor gas sensor. Sens. Actuators B Chem..

[59] Yamazoe N., Sakai G., Shimanoe K. (2003). Oxide semiconductor gas sensors. Catal. Surv. Asia.

[60] Ye A.H., Fan W.Q., Zhang Q.H., Deng W.P., Wang Y. (2012). CdS−Graphene and CdS−CNT nanocomposites as visible-light photocatalysts for hydrogen evolution and organic dye degradation. Catal. Sci. Tech..

[61] Yang K., Huang K., He Z.J., Chen X., Fu X.Z., Dai W.X. (2014). Promoted effect of PANI as electron transfer promoter on CO oxidation over Au/TiO_2_. Appl. Catal. B Environ..

[62] Zhang H., Lv X., Li Y., Wang Y., Li J. (2010). P25-graphene composite as a high performance photocatalyst. ACS Nano.

[63] Xiao F.X., Wang F.C., Fu X.Z., Zheng Y. (2012). A green and facile self-assembly preparation of gold nanoparticles/ZnO nanocomposite for photocatalytic and photoelectrochemical applications. J. Mater. Chem..

[64] Ding Y., Mo L.E., Tao L., Ma Y.M., Hu L.H., Huang Y. (2014). TiO_2_ nanocrystalline layer as a bridge linking TiO_2_ sub-microspheres layer and substrates for high-efficiency dye-sensitized solar cells. J. Power Sour..

