# Supplementary material for: Comparative Study of Two Different TiO2 Film Sensors on Response to H2 under UV Light and Room Temperature
Source: Sensors (Basel). 2016 Aug 8;16(8):1249. doi: 10.3390/s16081249 (PMC5017414; doi:10.3390/s16081249)
Supplement: Supplementary file 1 [file sensors-16-01249-s001.pdf]

Sensors **2016**, *16*, 1249; doi:10.3390/s16081249

Supplementary Materials: Comparative Study of Two Different TiO_2 Film Sensors on Response to H_2 under UV Light and Room Temperature

Xiaoying Peng, Zhongming Wang, Pan Huang, Xun Chen, Xianzhi Fu and Wenxin Dai

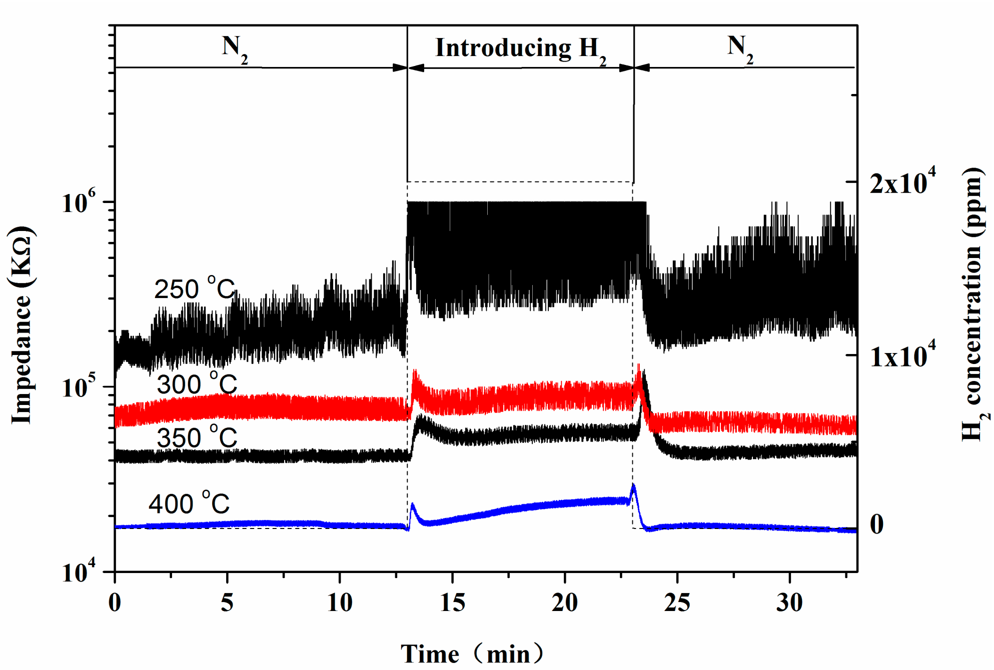

Figure S1. Gas sensing process to H_2 in N_2 atmosphere at different temperatures without UV light over TiO_2-I sample. The solid curves denote the impedance module of samples as function of time, and the dotted line denotes the concentration of H_2 during the testing process. However, the testing process at 450 °C could not be performed due to the temperature limit of the chamber.
